# 

**Published:** 1989-02

**Authors:** 


W1 BR323

104 NO.1 1989

D1---------SEO: B33740000

: BRISTOL MEDICO-CHIRURGICAL

JOURNAL

### BRISTOL MEDICO-CHIRURGICAL JOURNAL

VOLUME 104(i) FEBRUARY 1989

**Malformation, Mutation and Malignancy**

**Benefits of an Asthma Nurse**

**Hospital and Community Psychiatry**

**The Empoisoned Darts of Venus**

**New Dimensions in the N.H.S.**

**From our Correspondents**

**Reports from Societies**

NATIONAL LIBRARY
DEC 19 1989
OF MEDICINE

THE MEDICAL JOURNAL OF THE SOUTH WEST

